# Cardiorespiratory and metabolic determinants during moderate and high resistance exercise intensities until exhaustion using dynamic leg press: comparison with critical load

**DOI:** 10.1590/1414-431X20187837

**Published:** 2018-10-11

**Authors:** V.M. Arakelian, C.L. Goulart, R.G. Mendes, F.C. Caruso, V. Baldissera, R. Arena, A. Borghi-Silva

**Affiliations:** 1 Programa de Pós-graduação Interunidades em Bioengenharia, Universidade de São Paulo, São Carlos, SP, Brasil; 2 Laboratório de Fisioterapia Cardiopulmonar, Departamento de Fisioterapia, Universidade Federal de São Carlos, São Carlos, SP, Brasil; 3 Laboratório de Fisiologia do Exercício, Departamento de Ciências Fisiológicas, Universidade Federal de São Carlos, São Carlos, SP, Brasil; 4 Department of Physical Therapy, University of Illinois, Chicago, USA

**Keywords:** Load, Physiological, Muscle fatigue, Exercise, Cardiorespiratory

## Abstract

The objective of this study was to assess cardiovascular, respiratory, and metabolic responses during a commonly used dynamic leg press resistance exercise until exhaustion (T_Ex_) at different intensities and compare with critical load (CL). This was a prospective, cross-sectional, controlled, and crossover study. Twelve healthy young men (23±2.5 years old) participated. The subjects carried out three bouts of resistance exercise in different percentages of 1 repetition maximum (60, 75, and 90% 1RM) until T_Ex_. CL was obtained by means of hyperbolic model and linearization of the load-duration function. During all bout intensities, oxygen uptake (VO_2_), carbon dioxide production (VCO_2_), ventilation (V_E_), and respiratory exchange ratio (RER) were obtained. Variations (peak-rest=Δ) were corrected by T_Ex_. In addition, systolic and diastolic blood pressure (SBP and DBP), blood lactate concentration [La-] and Borg scores were obtained at the peak and corrected to T_Ex_. CL induced greater T_Ex_ as well as number of repetitions when compared to all intensities (P<0.001). During CL, Borg/T_Ex_, ΔSBP/T_Ex_, ΔDBP/T_Ex_, and [La-] were significantly lower compared with 90% load (P<0.0001). In addition, VO_2,_ VCO_2,_ V_E_, and RER were higher during CL when compared to 90 or 75%. T_Ex_ was significantly correlated with VO_2_ on CL (r=0.73, P<0.05). These findings support the theory that CL constitutes the intensity that can be maintained for a very long time, provoking greater metabolic and ventilatory demand and lower cardiovascular and fatigue symptoms during resistance exercise.

## Introduction

The prescription of intensity level for an exercise training program remains, to a certain degree, an art form, given the uncertainties and variabilities surrounding the association between exercise load and the cardiorespiratory response (1,2). The majority of these studies has been done in the aerobic training arena to develop precise methods for setting an exercise intensity at a level that can be sustained for a prolonged time without leading to fatigue (3).

Resistance exercise training has gained increasing recognition as a valuable component of a general exercise conditioning program (4). More recently, resistance training with increased volume of movement via increased numbers of sets and/or repetitions has shown a greater impact upon the lipid profile than increased intensity (5,6). In addition, significant positive changes can occur in cardiovascular and metabolic measurements after resistance training (7,8). For this reason, recent recommendations consider that dynamic moderate-intensity resistance training provides benefits in the general population and particularly in cardiac patients without causing additional risks, improving independence in activities of daily living, and, consequently, the quality of life (9).

However, little is known about the responses of acute resistance exercise of different intensities and if the load/time relationship, i.e., the critical load (CL), consistently could be considered as maximal lactate steady-state intensity and/or maximal intensity to be sustained by time (10 [Bibr B11]). CL is a theoretical construct whose mathematical definition refers to an external/mechanical power output (in watts) and is strongly determined by maximal oxygen uptake and associated with muscular aerobic power. This concept has been applied to many populations including athletes, cardiac patients, and elderly in different exercise modalities (10–12). The hyperbolic power has led to the development of several mathematical models, thereby providing a framework to more fully understand human endurance. For this reason, CL may provide a theoretical framework for prescribing both the intensity and duration of endurance-resistance exercise, representing thus the transition from heavy to severe intensity. This might be a novel approach to determine a sustainable intensity for resistance exercise, and have a new indicator for resistance exercise training programs (10).

However, currently, there is still shortage of information on cardiovascular and metabolic responses contrasting the CL with different loads (moderate and higher intensities) to train resistance exercise. In a previous study, we quantified the maximal load capacity above the aerobic critical load as a method for estimating anaerobic load capacity (10). However, cardiorespiratory and metabolic responses contrasting moderate and high resistance exercise with CL considering the time to exhaustion (T_Ex_) needs to be investigated.

Thus, the purpose of the present investigation was to compare cardiovascular, respiratory, and metabolic responses during a commonly used dynamic leg press resistance exercise to exhaustion at different intensities with CL. We hypothesized that CL may be considered as maximal sustained intensity over time on resistance exercise.

## Material and Methods

### Study design and subjects

This was a prospective, cross-sectional, controlled, and crossover study. Twelve apparently healthy and generally active males volunteered for this study. The subjects initially completed a health history questionnaire and bioimpedance analysis (Tanita Body Composition Analyzer - Model TBF 310, Japan) to determine body composition. All subjects signed a written informed consent prior to study initiation. The study was approved by the Ethics Committee of Universidade Federal de São Carlos (433/2008).

Prior to participation in the study, all volunteers underwent a clinical evaluation performed by a physician (cardiologist). This examination consisted of anamnesis, resting 12-lead electrocardiography, and a symptom-limited treadmill test (Bruce Protocol). Maximal oxygen uptake (VO_2max_) was determined by open circuit ergospirometry (Oxycon Mobile, Germany). Subjects were excluded if they were current smokers, taking any type of prescription medication, or experienced skeletal-muscle pain that would limit exercise performance.

### Procedures

The experiment was performed at the same time of day to minimize influences of circadian variations. The data collection environment was maintained at a room temperature between 22 and 24°C and relative air humidity between 40 and 60%. To ensure patient safety, blood pressure (BP) and electrocardiography (ECG) were monitored during all procedures.

### Exercise protocols

Subjects completed five resistance exercise assessments using the dynamic resistance leg press (LP) maneuver at a 45° angle (PL-48, Reforce, Brazil). The first test consisted of a one repetition maximum (1RM) test, and the next three were constant loads tests until exhaustion, in order to determine CL, and a final test to exhaustion at the CL intensity. At least 48 h rest was allowed between each assessment. All subjects underwent each load on separate occasions at a similar time of the day, for determination of test-retest reliability and validity. The intra-class correlation coefficients for these repeated assessments ranged from 0.93 to 0.95, indicating excellent reliability. To reduce bias and possible effects of motivation, subjects were not informed of their progress during any of the testing procedures.

### One repetition maximum test (1RM)

A gradual increase in resistance was applied until the individual could not correctly perform more than one repetition of the LP exercise. The volunteer was placed in a seated position on the device and during the movement cycle performed knee and hip flexion by eccentric contraction of the quadriceps muscles and the gluteus, until reaching an angle of 90° between the thigh and leg, and then returned to the starting position through concentric contraction.

Based on the protocol for determining the maximal load (13), we performed a modified protocol, which consisted of: *i*) general warm-up on a cycle ergometer, *ii*) a 10 repetitions warm-up on the dynamic LP resistance exercise, at an extremely low load (20 kg), which was also used to verify the execution technique, and *iii*) applying a load approximating 1RM. If the first 1RM load was insufficient, a new attempt was made following a 5-min recovery period. If the subject could not perform a full repetition during the first 1RM attempt, a new attempt was made at the same weight after a 5-min rest period. If the repetition was not completed on the second attempt, another attempt at a lower load was performed. The highest load successfully completed was defined as the 1RM (14). The 1RM was determined within 5 attempts for all subjects, as described by our group's previous study (10).

### Three constant load tests to determine CL

**Figure 1.**
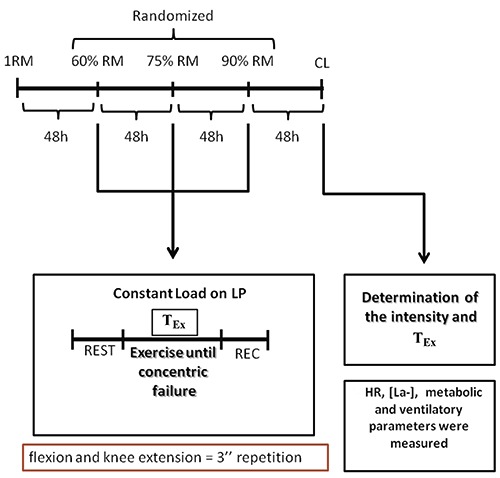
Flowchart of the study. RM: repetition maximum; REC: recovery; CL: critical load; LP: Leg press; T_Ex_: time to exhaustion; HR: heart rate; [La^-^]: lactate.

This protocol was applied to evaluate the cardiorespiratory responses and blood lactate concentration [La^-^] at three different intensities based upon each subjects 1RM. Following a warm-up period, each of the three loads was performed to concentric failure, and, in random order, in different days (with a rest period of 48 h). Randomization was made by drawing of shuffled, opaque, coded envelopes that were opened immediately before starting the first intensity. All subjects were blinded of the load (intensity of RM) that was executed and one investigator who did not participate in the data collection included the loads in the leg press and the intensity determined for that test. The following intensities were used for these constant load maneuvers to determine CL: *A*) 60% 1RM; *B*) 75% 1 RM; *C*) 90% 1 RM (Figure 1).

The participants performed one or two sessions of adaptation on LP (Leg Press 45°, Reforce, Brazil) to establish the correct biomechanics of the movement. After the familiarization period, in starting on another day, the different intensities were applied in random order on different days, as follows: *i*) each repetition cycle lasted about 3 s, with 1.5 s for the concentric phase and 1.5 s for the eccentric phase, controlled by a metronome; *ii*) motion knee angle between 90 and 180 degrees, controlled by an electrogoniometer (15) (EMG system, Brazil).

The cardiovascular responses were assessed by HR, BP, and ECG recording. The metabolic response was assessed breath-by-breath with a ventilatory expired gas analysis (Oxycon Mobile). The beginning of the test was conducted with prior calibration of the gas analysis system using a 3-liter syringe and standard calibration gases (16% O_2_ and 4% CO_2_). Data was recorded two min before the exercise assessment and considered to be baseline values for minute ventilation (V_E_), oxygen uptake (VO_2_), carbon dioxide production (VCO_2_), and respiratory exchange ratio (RER). The peak values were defined as the highest 15-s averaged VO_2_ value of the last 30 s of the RE. All these variables were assessed for an additional 5-min recovery. Cadence of movement in all intensities was controlled by electromyographic representation of the vastus medialis muscle and the angulation in degrees by an electrogoniometer (10). T_Ex_ at all intensities was defined as the inability to continue the contractions due to leg fatigue or difficulty to maintain the movement cadence and/or movement amplitude.

In addition, 25 µL of arterial blood was collected through punctures in the ear lobe to measure [La^-^], one collected before the beginning of the test, another 30 s after the test, and after 5 min of recovery (YSI 1500, Sport, USA) (16). The highest values were considered for analysis (10).

### Determination and assessment of CL

**Figure 2.**
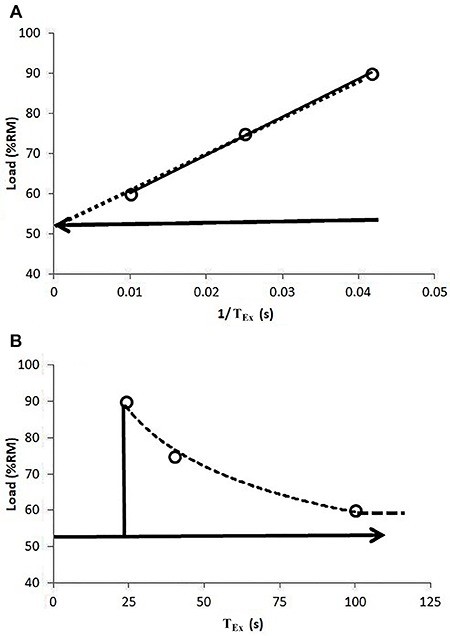
The load-time relationship in response to three progressive resistance exercise tests for all subjects. *A*, Linearized response as a function of percentage peak load. *B*, A hyperbolic relationship was found in all subjects. The asymptote represents the critical load. RM: repetition maximum; T_Ex_: time to exhaustion

The final resistance exercise assessment was performed on a different day, in order to determine CL tolerability, which was determined by rating the perceived exertion at the end of the exercise (Borg scale). The CL was determined by linear regression of the points made during the construction of the graph: load × inverse of time (time = duration of exercise until fatigue, Figure 2). This load was observed at the highest sustainable work rate, i.e., the CL, also held to volitional fatigue. During this final assessment, HR, BP, ventilatory expired gases, and blood samples were collected as in the tests described previously.

### Monitoring during the exercise protocols and criteria for discontinuing the tests

To minimize risks of complications and identify test termination criteria (17), subjects were monitored by the MC5 electrocardiographic (ECG) lead (monitor Ecafix TC500), in which behavior was evaluated for possible ischemic changes, and based on previous studies (18,19), to identify the presence and quantity of arrhythmias during and after the different exercises.

Blood pressure was measured during rest and at the end of each intensity by the indirect auscultatory method using a sphygmomanometer (Welch Allyn Tycos, USA) and a stethoscope (Littmann, Master Cardiology, 3M, USA).

### Data analysis and statistics

Statistical analyzes were performed using the Statistic software package (version 8, StatSoft, Inc., USA). Data are reported as means±SD. The variables demonstrated a normal distribution, justifying the use of parametric tests (Shapiro-Wilks). Leneve's test was used to demonstrate the homogeneity of variances. The Δ were obtained by calculating peak – rest / time to fatigue for each intensity. The parameters Borg, ΔSBP, ΔDBP, [La^-^], ΔV_E_, ΔVO_2,_ ΔVCO_2_, and ΔRER were corrected to T_Ex_ and analyzed through one-way ANOVA. When a significant F ratio was obtained, the Tukey's *post hoc* test was used for all comparisons. Pearson's coefficient was used to determine correlations between variables. All tests with a P<0.05 were considered statistically significant.

## Results

All assessments were well tolerated without inappropriate response warranting test termination. Subject characteristics are listed in Table 1, including VO_2max_, maximum load during the 1RM test, and CL. This value represented approximately 53% 1RM (Figure 2). Thus, it was possible to see the load-time relationship in response to three progressive resistance exercise tests (Figure 2A) and a well-characterized rectangular hyperbolic (Figure 2B). In addition, the load-time relationship and hyperbolic characteristics were confirmed by the excellent linear fits (0.93–0.99).

**Table 1.** Characteristics of the subjects.

Parameters	Volunteers (n=12)
Age (years)	23±3
Body mass (kg)	79.2±9.9
Height (cm)	1.8±0.1
BMI (kg/cm^2^)	24.7±2.9
FP (%)	17.2±5.3
FBM (kg)	13.6±5.2
LBM (kg)	64.9±5.6
VO_2max_ (mL·kg^-1^·min^-1^)	35.6±6.1
Max Load (kg) -1RM	297.1±44.1
CL (%)	52.5±4.9
R^2^	0.9917

Data are reported as means±SD. BMI: body mass index; FP: fat percentage; FBM: fat body mass; LBM: lean body mass; VO_2max_: maximal aerobic capacity; Max Load: 1 repetition maximum (RM) test obtained in leg press at 45°; CL: critical load; R^2^: coefficient of determination.

**Figure 3.**
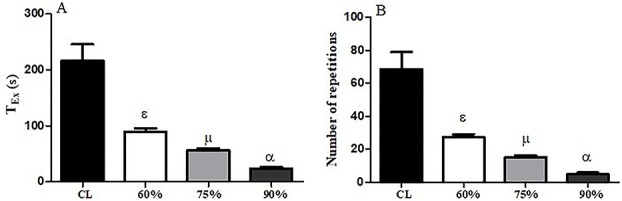
*A*, Time until exhaustion (T_Ex_) and *B*, number of repetitions performed in the four intensities during resistance exercise. CL: critical load. Data are reported as means±SD. ^ε,µ,α^P<0.05 compared to CL (one-way ANOVA).

Figure 3 shows a comparative evaluation between all loads and CL in relation to the time until exhaustion and the number of repetitions performed. We observed that the number of repetitions and the time until exhaustion were greater at CL when compared with all intensities (Figure 2, P<0.05).

**Figure 4.**
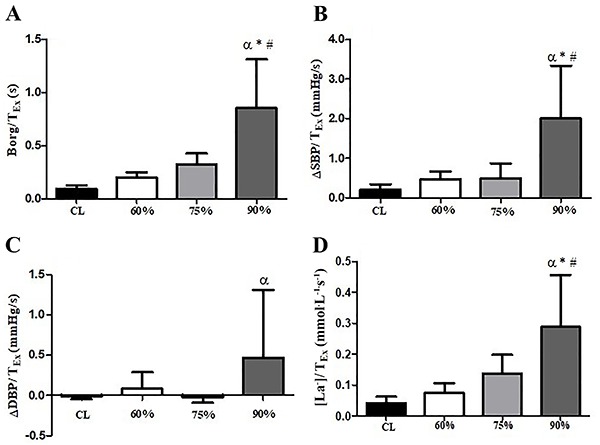
Peak Borg scale corrected to time to exhaustion (T_Ex_) (*A*), variation of systolic blood pressure (ΔSBP) corrected to T_Ex_ (*B*), variation of diastolic blood pressure (ΔDBP) corrected to T_Ex_ (*C*), and lactate ([La-]) corrected to T_Ex_ (*D*) responses at different intensities as well as on critical load (CL). Data are reported means±SD. SBP. ^α^P<0.05 compared to CL; *P<0.05 compared to 60%; ^#^P<0.05 compared 75% (ANOVA).

This study investigated the physiological determinants, such as blood pressure and chronotropic responses as well as blood lactate concentration and RPE during all intensities, compared to CL (Figure 4). Peak Borg scale, SBP, DBP, and [La^-^] did not differ between intensities. However, Borg scale, ΔSBP and DBP, and [La^-^] corrected to T_Ex_ were significantly lower during CL compared with 90% load (P<0.0001).

**Figure 5.**
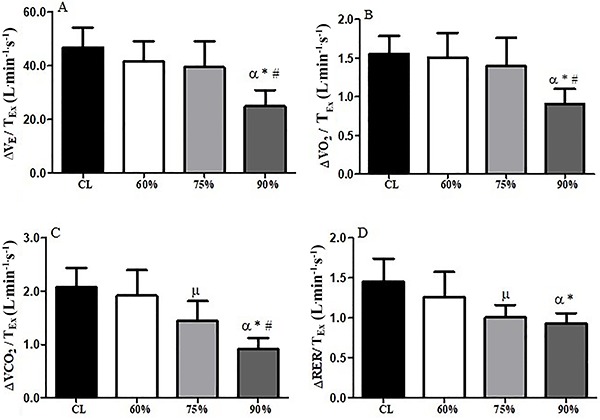
*A*, Minute ventilation variability (ΔV_E_), *B*, oxygen uptake variability (ΔVO_2_), *C*, carbon dioxide production variability (ΔVCO_2_), and *D*, respiratory exchange ratio variability (ΔRER) corrected to time to exhaustion (T_Ex_) at different intensities as well as on critical load (CL). Data are reported means±SD. ^α^P<0.05 compared to CL; *P<0.05 compared to 60%; ^#^P<0.05 compared to 75%; ^µ^P<0.05 compared to CL (ANOVA).

**Figure 6.**
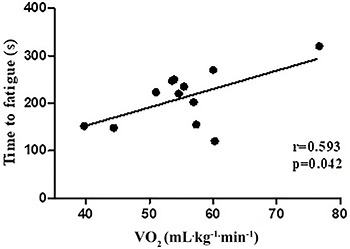
Pearson correlation between fatigue time and oxygen uptake (VO_2_) during critical load.

Peak V_E_, VO_2,_ VCO_2_, and RER did not differ between intensities. However, comparing the ΔV_E_, ΔVO_2,_ ΔVCO_2_, and ΔRER corrected to T_Ex_ during the four intensities of resistance exercise on leg press, greater values were observed during CL when compared with all phases (P<0.0001). In addition, the ΔVCO_2_ and ΔRER corrected to T_Ex_ were significantly higher during CL compared with 75% load (Figure 5). The T_Ex_ was significantly correlated with VO_2_ obtained during the CL intensity (r=0.593, P=0.042) (Figure 6).

## Discussion

### Main findings of this study

The present study confirmed the existence and the intensity of CL by the hyperbolic and linear model during LP resistance exercise, which is characterized as the maximum load that can be sustained by the time (10,19). CL could be used to establish the maximum training intensity (load in percentage of 1RM – approximately 54%). Additionally, we investigated the physiological determinants of resistance capacity, using a dynamic LP resistance exercise until exhaustion. The main original findings of the present study can be summarized as follows: *i*) CL promoted greater exercise time and number of repetitions; *ii*) these findings were consistent with lower cardiovascular, blood pressure, and blood lactate concentration responses, as well as subjective responses and greater ventilatory and oxygen uptake responses when corrected for time until exhaustion; and finally *iii*) time until fatigue was strongly correlated to oxygen uptake during CL.

### Theory concept of critical load

Several studies have examined the concept of critical power (CP) and time to fatigue, primarily during aerobic or endurance exercises, such as cycle ergometry, treadmill, or swimming (20–24). Conversely, few previous investigations have reported the physiological determinants during exercise (3,16,25,26). In relation to resistance exercises, to our knowledge, this is the first study to investigate the existence of a CL using a lower extremity resistance exercise. Our findings may imply that CL existence corresponds to approximately 54% of 1RM in healthy young adults. However, this concept is not new in CL, since during isometric maximal contractions of the quadriceps, Burnley et al. (27) observed a "critical torque" during a series of submaximal intermittent tests.

According to Arakelian et al. (10) and Vanhatalo et al. (28), there are three behavior patterns in dynamic physical exercise that can be distinguished by the kinetic behavior of some physiological determinants during exercise and CP would represent the transition from heavy to severe intensity. However, the concept of CP is contradictory to some authors. Brickley et al. (19) examined the physiological responses of young men trained on a cycle ergometer at the intensity of CP, which was being assessed by time to exhaustion via VO_2_, HR, and [La^-^]. These authors reported that CP does not represent a steady state because there is an increase in VO_2_, VCO_2_, [La^-^], and HR. Moreover, according to these authors, the intensity of CP reached during this type of aerobic exercise was determined to be approximately 80% of VO_2max_. In this context, the authors redefined CP as being the highest exercise intensity in a non-steady state, which can be maintained for 20 to 40 min duration.

Moreover, Bull et al. (25) determined that at the estimated critical velocity, physical activity could not be sustained for more than 60 min. Additionally, previous studies have shown approximately 18–60 min (10,29–31), for the exhaustion time at CP; however, an increase of [La-] during exercise (3,19) was observed. In our study, we concluded that to estimate CL during a leg press dynamic resistance exercise protocol, the endurance time was approximately 120 s, corresponding to approximately 35 repetitions at an intensity of 54% of 1RM.

### Physiological responses at all intensities contrasting with critical load

In the present study, the intensity was obtained through linear and hyperbolic regression by time, and we observed that, despite that the resistance exercise was prolonged until exhaustion, fatigue symptoms, blood pressure, and blood lactate concentrations were the lowest when compared with all other intensities. However, V_E_, VO_2_, VCO_2_, and RER variations were highest during resistance exercise at CL, indicating higher ventilatory and metabolic demand in this kind of exercise intensity. These results were striking and can be explained by the fact that the execution time and the number of repetitions allowed greater ventilatory and oxygen uptake responses during the resistance exercise, when it was maintained at this moderate intensity and for a prolonged time. In this context, to our knowledge, this is the first study to evaluate ventilatory demand and oxygen uptake at resistance exercise, especially on CL. Our results are similar to those of De Sousa et al. (32) who showed that lower loads, as constant-load resistance exercise at 30% RM, corresponded to a steady-state of ventilatory, cardio-metabolic parameters, and ratings of perceived exertion. The present study, considering a new approach (CL), indicated that moderate resistance intensity could enhance ventilatory and metabolic responses. These results have relevance since they could indicate that resistance exercise protocols of training, considering higher volumes (number of repetitions until exhaustion in lower intensities), may enhance aerobic performance, as well as have the advantages of conventional resistance training.

In relation to physiological responses obtained in purely dynamic protocols, Bull et al. (25) evaluated the HR, VO_2,_ VCO_2_, and [La^-^] on critical velocity (CV). Their results suggested the CV did not represent the maximum speed that could be maintained indefinitely without fatigue for most people, because the estimated CV represented 96% of HR_max_, 82% of VO_2max_, and [La^-^] between 5 and 8.2 mmol/L. In the present study, similarly to the above-mentioned study, [La^-^] at CL achieved peak values of 7.3±2.1 mmol/L. Dekerle et al. (33) verified that CP is a potential indicator of the maximum [La^-^] steady state in trained men. They concluded that CL is a maximum lactate steady state (MLSS), since there is an increase in [La^-^] that can be sustainable over time.

### Methodological considerations

Similar to the present study, some authors (10,19) believe that as CL is estimated by mathematical models based on the inverse ratio load-time, or even the asymptote of the load-time relationship, it can represent the intensity that theoretically can be maintained for a long time. In this context, it is known that due to factors such as substrate depletion, temperature regulation and body fluid, and electrolyte balance even at high intensities (above anaerobic threshold) there may be a contribution to fatigue during prolonged exercise, and thus the mathematical expression is unlikely to provide an accurate representation of an infinitely sustainable load (8,33). Besides, as the protocol is continuous, unlike what happens in the practice of resistance exercise, which is accomplished through series and intervals, it cannot be maintained for an extended period.

Another factor that should be taken into account in the current study is the validity of the protocol used to determine CL, as this index is protocol-dependent (34). In addition, several studies showed that CP is dependent on the duration of exercise and loads selected to predict it (35). Accordingly, the loads selected for the determination of CL or CP should allow the attainment of VO_2max_ during constant load exercise, i.e., T_Ex_ between two and 15 min (36). However, in our study, we could not achieve the supra-maximal intensities, since it is impracticable during resistance exercise.

In most protocols used in the literature to evaluate CP, exhaustion tests are performed with control of cadence movements (8,30), as in our study. However, in the research of McLellan et al. (32) and Dekerle et al. (37), the cadence was chosen by each volunteer, i.e., they were instructed to pedal at a self-selected pace. Some investigators suggest that the spontaneity of rhythm has a strong relationship with cardiorespiratory parameters or with neuromuscular fatigue (37), thus the duration of each session (in the case of cycle ergometer) can influence the measurement of CP. Nevertheless, Dekerle et al. (37) did not report a significant difference in average value of the pedaling cadence compared between the tests, but differences among the volunteers might have been because each one chose their own rhythm employed during each test. Thus, the effect on movement control may be directly related to the methodology used and the parameters determining the relationship hyperbolic load versus time; results between studies are difficult to compare (26).

As another important methodological consideration, the concept of CL needs to be investigated in other types of resistance exercises to compare different responses. In addition, it is important to note that there are physiological differences in fitness level, training status, and predisposed genetic differences in muscle fiber types, all impacting approaches to exercise prescription in a unique way and thus, creating the potential for variability in CL responses.

This study emphasizes the practical importance of dynamic resistance exercise and its load-time relationship, which may add methodological evidence for therapeutic interventions aimed at optimally rehabilitating and restoring strength and/or muscular endurance.

These findings support the theory that CL constitutes the intensity that can be maintained for a very long time, provoking great metabolic and ventilatory demand and low cardiovascular and fatigue symptoms during resistance exercise.
